# The CRISP theory of hippocampal function in episodic memory

**DOI:** 10.3389/fncir.2013.00088

**Published:** 2013-05-06

**Authors:** Sen Cheng

**Affiliations:** ^1^Mercator Research Group “Structure of Memory”, Ruhr-Universität BochumBochum, Germany; ^2^Faculty of Psychology, Ruhr-Universität BochumBochum, Germany

**Keywords:** hippocampus, episodic memory, pattern completion, pattern separation, CA1, CA3, dentate gyrus, standard model

## Abstract

Over the past four decades, a “standard framework” has emerged to explain the neural mechanisms of episodic memory storage. This framework has been instrumental in driving hippocampal research forward and now dominates the design and interpretation of experimental and theoretical studies. It postulates that cortical inputs drive plasticity in the recurrent cornu ammonis 3 (CA3) synapses to rapidly imprint memories as attractor states in CA3. Here we review a range of experimental studies and argue that the evidence against the standard framework is mounting, notwithstanding the considerable evidence in its support. We propose CRISP as an alternative theory to the standard framework. CRISP is based on Context Reset by dentate gyrus (DG), Intrinsic Sequences in CA3, and Pattern completion in cornu ammonis 1 (CA1). Compared to previous models, CRISP uses a radically different mechanism for storing episodic memories in the hippocampus. Neural sequences are intrinsic to CA3, and inputs are mapped onto these intrinsic sequences through synaptic plasticity in the feedforward projections of the hippocampus. Hence, CRISP does not require plasticity in the recurrent CA3 synapses during the storage process. Like in other theories DG and CA1 play supporting roles, however, their function in CRISP have distinct implications. For instance, CA1 performs pattern completion in the absence of CA3 and DG contributes to episodic memory retrieval, increasing the speed, precision, and robustness of retrieval. We propose the conceptual theory, discuss its implications for experimental results and suggest testable predictions. It appears that CRISP not only accounts for those experimental results that are consistent with the standard framework, but also for results that are at odds with the standard framework. We therefore suggest that CRISP is a viable, and perhaps superior, theory for the hippocampal function in episodic memory.

## INTRODUCTION

The human hippocampus is important for episodic memories (Scoville and Milner, 1957). However, despite the enormous research effort invested since the initial discovery, the neuronal mechanisms underlying episodic memory storage and recall remain unclear. To understand the role of the hippocampus, or any other brain region, we need a theory for its computational function and for how the neural network in that region gives rise to this function (Levy, 1989). Of the many theories that have been proposed for hippocampal function, one stands out. It originated with Marr (1971) and was refined by several authors over four decades (McNaughton and Morris, 1987; Buzsaki, 1989; Treves and Rolls, 1992, 1994; O’Reilly and McClelland, 1994; McClelland et al., 1995; Becker, 2005; Wiskott et al., 2006). This theory has dominated the field to such an extent that Nadel and colleagues termed it the “standard model” (Nadel and Moscovitch, 1997; Nadel et al., 2000). We prefer to use the term “standard framework” to emphasize that it is a collection of assumptions that have accumulated over the years, not a unitary model. In our opinion, major advances in experimental techniques have recently yielded contradicting results. Many of these results have been misinterpreted as supporting the standard framework, perhaps, due to the lack of a viable alternative theory.

Here we propose a new theory for the hippocampal function in episodic memory storage: CRISP. The crucial elements of CRISP are Context Reset by the dentate gyrus (DG), Intrinsic Sequences in cornu ammonis 3 (CA3), and Pattern completion in cornu ammonis 1 (CA1). It integrates components that individually have been discussed before, but never together in a coherent framework. To store episodic memories, sequences of external stimuli are mapped onto pre-established intrinsic sequences of neural activity in CA3, not imprinted onto the plastic CA3 recurrent network. CA1 performs pattern completion to compensate for any distortions that were introduced in the recall of sequential elements in CA3. DG overrides the intrinsic CA3 dynamics to initiate recall and to enable the storage of novel sequences that are similar to, but distinct from, previously stored ones. We argue in this article that CRISP yields a more consistent interpretation of the major results in hippocampal research than the standard framework does.

The focus of this article is episodic memory, but we need to address its relationship to the prominent representation of space, which is also found in the hippocampal formation. The discovery of place cells in the rodent hippocampus (O’Keefe and Dostrovsky, 1971) and grid cells in the rodent medial entorhinal cortex (MEC; Hafting et al., 2005) have spawned a great number of studies into the nature of, and neural mechanisms underlying, these spatial representations. For instance, the rodent hippocampus was found to be required for spatial learning (Morris et al., 1982) and there is consensus that the hippocampus in humans is crucial for both episodic as well as spatial memory (Eichenbaum et al., 1999; Burgess et al., 2002). Nevertheless, questions remain about the precise relationship between episodic and spatial representations in different species. For instance, Eichenbaum et al. (1999) suggest that episodic memory is the primary function of the hippocampus and spatial information is but one aspect thereof. By contrast, O’Keefe and Nadel (1978) argue that a cognitive map evolved in the hippocampus of non-human mammals to support spatial navigation and that this cognitive map is used in humans to support episodic memory. We cannot give a full account of this controversy in this article, and note that both the standard framework and CRISP do not make assumptions about how the episodic memory system evolved. Spatial information can therefore be part of the encoded memory pattern and in fact many electrophysiological studies cited in this article are recordings from place cells.

In the next section, we will first introduce the standard framework and discuss the experimental evidence in favor of and against the standard framework. In Section “CRISP: A New Theory for Hippocampal Function,” we will introduce our new theory and, then in Section “Experimental Support for the Crisp Theory,” discuss how it can account better for the experimental data. In Section “Comparison to Other Theories,” we will briefly compare our new theory to other theories of hippocampal function and then conclude.

## THE STANDARD FRAMEWORK

The starting point in the standard framework is the realization that the successive storage of patterns in a neural network leads to catastrophic interference (McCloskey and Cohen, 1989), i.e., the storage of a new pattern can destroy previously stored patterns. The standard framework postulates that two complementary memory systems, the neocortex and the hippocampus, have evolved to solve this problem in two different ways (McClelland et al., 1995). The so-called two-stage model postulates that memories are first stored in the hippocampus and then gradually transferred to the cortex (Wickelgren, 1979; Teyler and DiScenna, 1986; Buzsaki, 1989; Gluck and Myers, 1993; Alvarez and Squire, 1994). This transfer is thought to be facilitated by repeated replay of the activity patterns stored in hippocampus (Buzsaki, 1989; McClelland et al., 1995).

In the standard framework, area CA3 is the core of the hippocampal memory system; it stores memories in an auto-associative memory network formed by its recurrent connections (Marr, 1971; McNaughton and Morris, 1987; O’Reilly and McClelland, 1994; Treves and Rolls, 1994). CA3 is usually envisioned to operate like a Hopfield net (Hopfield, 1982), which stores memory patterns as attractor states. The network dynamics retrieves the full memory pattern when a partial or noisy cue is provided (pattern completion). Since similar patterns that represent different memories would interfere in CA3, DG is thought to be a preprocessing stage that maps similar input patterns to distinct CA3 patterns (Marr, 1971; McNaughton and Morris, 1987; O’Reilly and McClelland, 1994; Treves et al., 2008). This pattern separation process is facilitated by the sparse activity in DG, its sparse but strong projections to CA3, and synaptic plasticity. Recently, it was suggested that pattern separation is further aided by adult neurogenesis in DG, a process that provides new granule cells that arguably have little overlap with older DG cells with respect to their projections to CA3 (Becker, 2005; Wiskott et al., 2006; Aimone et al., 2009).

### INSUFFICIENT EVIDENCE FOR TWO MEMORY SYSTEMS WITH DIFFERING TIMECOURSE OR STABILITY OF MEMORIES

The foundation of the standard framework rests on the existence of two memory systems; a hippocampus that can store memories rapidly and neocortex that cannot because of catastrophic interference. A consequence of this view is that declarative memories are stored initially in the hippocampus and then transferred slowly to neocortex for long-term storage. These premises are both supported by experimental evidence. First, the hippocampus is important in several one-trial learning paradigms such as the working memory version of the water maze (Steele and Morris, 1999; Nakazawa et al., 2003), one-trial pair associate learning (Day et al., 2003), short exposure contextual fear conditioning (Cravens et al., 2006), and extinction training after trace conditioning (Kishimoto et al., 2006). These observations indicate that the hippocampus supports rapid memory storage. Second, memories that are initially dependent on the hippocampus gradually become independent of the hippocampus in a process called systems consolidation. More specifically, remote memories are less affected by hippocampal damage than recent memories are. Such graded retrograde amnesia has been observed in several species after insult to the hippocampus (Scoville and Milner, 1957; Sagar et al., 1985; Winocur, 1990; Zola-Morgan and Squire, 1990; Kim and Fanselow, 1992). These observations support the hypothesis that memories are transferred from the hippocampus to the neocortex.

However, in contradiction to the standard framework, evidence suggests that the speed and stability with which hippocampus and neocortex store memories are not qualitatively different. For instance, rapid learning can occur in the absence of hippocampus as well. Hippocampal patients show one-trial learning of associations (Murray and Mishkin, 1985; Vargha-Khadem et al., 1997), and faces (Cipolotti et al., 2006; Bird et al., 2008), and rapidly acquire novel semantic memory through fast remapping (Sharon et al., 2011). These studies indicate that the neocortex can support rapid learning in the absence of a hippocampus, and it remains unexplained in the standard framework why neocortex does not suffer catastrophic interference in these cases, as it does in the case of episodic memory.

Secondly, the standard framework cannot account for the instability of supposedly consolidated memories in the neocortex and for many results on retrograde amnesia. Even consolidated memories require active maintenance in neocortex for long-term stability (Shema et al., 2007), and interestingly and when a consolidated memory is retrieved, it becomes dependent on the hippocampus again (reconsolidation; Misanin et al., 1968; Debiec et al., 2002). A mounting number of studies find that even remote episodic-like memories are lost after hippocampal damage, e.g., in spatial tasks (Bolhuis et al., 1994; Martin et al., 2005) and contextual fear conditioning (Lehmann et al., 2006; Sutherland et al., 2008), and that retrieval of remote episodic memories engages the hippocampus in humans (Nadel et al., 2007; Ryan et al., 2010; Harand et al., 2012). Some authors suggest that remote episodic memories might only appear to be spared in amnesics because they retrieve remote autobiographical information based on semantic, rather than episodic, memories (Nadel et al., 2000; Steinvorth et al., 2005). Taken together, these studies suggest that memories are not always stored first in the hippocampus and then transferred to the cortex. Neither are they always more stable in the neocortex than in the hippocampus. Rather, they differ in some other fundamental aspect

### PLASTICITY IN THE RECURRENT CA3 IS NOT NECESSARY FOR RAPID LEARNING

In the standard framework, CA3 is crucial for rapidly storing associations in its recurrent network. Hence, one would expect that plasticity in CA3 is essential for hippocampally dependent one-trial learning and CA3 responses change significantly and rapidly during learning. Indeed, in the absence of *N*-methyl-D-aspartate (NMDA)-mediated plasticity in CA3, animals had deficits in a one-trial learning task in the Morris water maze, in which a novel platform location was used every day (Nakazawa et al., 2003). Along similar lines, Roth et al. (2012) found that CA3 place cell firing properties changed more rapidly than CA1.

However, upon closer examination the experimental evidence does not establish the necessity of plasticity in the recurrent CA3 synapses for rapid, one-trial learning. For instance, the one-trial learning deficit cited in the above paragraph was found in a late block of training (13–17 days), but not on days 5–12 (Nakazawa et al., 2003). A similarly complex picture arose when the same CA3-NMDA mutants were tested in single-trial contextual fear conditioning (Cravens et al., 2006). Mutants showed a deficit when tested 3 h after conditioning, but performed as well as controls when tested after 24 h. While this and the previous study suggest a role of plasticity at the recurrent CA3 synapse in the learning of some aspects of the tasks, they argue against the notion that plasticity in CA3 is required for single-trial memory storage *per se* because mutants were able to store and retrieve one-trial memories in some conditions. This hypothesis is further supported by another experiment involving the CA3-NMDA knockout mice. In trace eyeblink conditioning, mutants learned the association as well as controls did (Kishimoto et al., 2006), but were impaired during extinction learning.

Contrary to what we would expect if new memories were imposed on and stored in CA3, neural responses in CA3 do not appear to change significantly and suddenly when new items or situations are encountered. Since animals can form memories in one-trial learning experiments in 30 s or less (Nakazawa et al., 2003), we expect significant changes on the time scale on the order of seconds. A number of studies have recorded from the hippocampus while animals learn about novel environments (Wilson and McNaughton, 1993; Frank et al., 2004; Lee et al., 2004a) and observed small refinements of place fields such as changes in rate and shape, but no large shifts of place field locations or rearrangements of relative place fields. Also, these changes occurred on the time scale of minutes; other studies measure the rate of change on even longer timescales of tens of minutes (Leutgeb et al., 2004) or days (Roth et al., 2012). It appears that these changes cannot form the basis for episodic memory formation.

Some studies even suggest that neural responses in CA3 do not change significantly at all during learning, while they do in CA1. This pattern was observed in electrophysiological recordings during learning of new goal locations in a familiar environment (Dupret et al., 2010) and during exploration of a novel environment (Karlsson and Frank, 2008). Further evidence for experience-dependent changes in neural activity in CA1, rather than CA3, come from studies of the immediate-early-gene for activity-regulated cytoskeleton-associated (ARC) protein, an immediate-early-gene related to synaptic plasticity. In CA3, a constant fraction of cells expressed ARC throughout learning, but in CA1, a monotonically increasing fraction of cells expressed ARC (Miyashita et al., 2009). These results therefore strongly suggest that the hippocampus plays a role in one-trial learning, but apparently not because memories have to be stored rapidly in area CA3.

### THE CASE AGAINST AUTO-ASSOCIATIVE MEMORY STORAGE IN RECURRENT CA3

The suggestion of an auto-associative memory system in CA3 leads to at least two functional predictions. First, similar patterns within the same basin of attraction are lumped together, while dissimilar patterns are split across different attractors and become more distant (O’Reilly and McClelland, 1994; McClelland and Goddard, 1996; Guzowski et al., 2004). Second, CA3 is required for memory recall based on a partial cue (pattern completion). In support of the first prediction, several studies found that similar environments induce nearly identical activity patterns of place cells activity in CA1 and CA3, whereas distinct environments induce quite different activity patterns (Lee et al., 2004b; Leutgeb et al., 2004, 2007; Vazdarjanova and Guzowski, 2004; Wills et al., 2005). A recent study found that one ensemble representation can switch to another rapidly within a single theta cycle (Jezek et al., 2011). Regarding the second prediction, experimental studies have argued that pattern completion tasks require CA3 and plasticity in the recurrent CA3 synapses (Nakazawa et al., 2002; Gold and Kesner, 2005; Fellini et al., 2009). In these tasks, animals were trained in the water maze while four prominent visual cues were present and later tested with a subset of these cues, ranging from zero to four cues. Animals with CA3 lesions or impaired plasticity in CA3 showed no deficit during retrieval when all four spatial cues were available during testing, but were severely impaired when only one or two of the training cues were present. The authors of these studies argue that animals had to rely on pattern completion to fill in the missing cues and that this process requires CA3 and plasticity in the recurrent CA3 synapses.

There are significant issues with both lines of evidence that support auto-associative memory in CA3. First, the discontinuous representation of similar vs. dissimilar environments appears to be an artifact of the training protocol rather than the necessary result of attractor dynamics in CA3 (Colgin et al., 2010). When trained differently, the population vector differed continuously for incremental changes in the environment (Leutgeb et al., 2005; Colgin et al., 2010). Second, in the standard framework, CA3 is thought to perform pattern completion because it is thought to store memory patterns in an auto-associative manner. However, neither plasticity in CA3 nor CA3 itself seem necessary for memory storage, as demonstrated by experiments in which animals with CA3 impairment can successfully recall the location of the platform in the water maze when all training cues are available (Nakazawa et al., 2002; Gold and Kesner, 2005; Fellini et al., 2009). In the cue-impoverished conditions, the deficit clearly seems to be in retrieval. Since animals with lesions of the entire hippocampus show learning deficits in similar experiments (Morris et al., 1982; Gilbert et al., 1998), these studies together suggest that memory storage must occur in parts of the hippocampus outside of CA3. In summary, in our opinion there is no firm experimental evidence for the existence of an auto-associative memory store in CA3.

### UNEXPLAINED SEQUENTIAL ACTIVITY IN THE OFFLINE STATE

In the two-stage model, replay of prior neural activity patterns is the neural mechanism for memory transfer between hippocampus and neocortex (Buzsaki, 1989; McClelland et al., 1995). Repetitive replays could allow for cortical learning without the danger of catastrophic interference (McCloskey and Cohen, 1989). Indeed, place cells fire spikes in offline states, such as sleep or wakeful quiescence, in the same or reverse sequence as during active exploration (Lee and Wilson, 2002; Foster and Wilson, 2006; Diba and Buzsáki, 2007). These replay events are frequently associated with ripples, brief high-frequency bursts in the local field potential, initiated in CA3 and propagated to CA1 (Ylinen et al., 1995; Nakashiba et al., 2009). Many experimental observations on replay in the hippocampus are consistent with the standard framework. For instance, the frequency of reactivation correlates with the duration of earlier experiences (O’Neill et al., 2008) and, importantly, with the subsequent performance on memory tasks (Axmacher et al., 2008; Dupret et al., 2010). And disruption of ripples, and thus reactivation, during sleep impairs spatial learning (Girardeau et al., 2009).

However, the assumption of the standard framework that hippocampal assemblies representing episodic memories are imprinted by external inputs is difficult to reconcile with recent experimental findings on offline sequential activity (OSA) in the awake state. Awake OSA was found to be important for spatial learning (Jadhav et al., 2012). However, awake OSA does not simply reflect the properties of sensory-driven activity that occurred earlier (for a review, see Buhry et al., 2011). In place cells representing a novel part of an environment, reactivation was stronger than in cells representing a familiar part (Cheng and Frank, 2008), even though the novel experience had much less time to imprint activity patterns on the hippocampus. Trajectories that the animal had never traveled were replayed, and the distribution of replay intervals suggests that they were independent of the preceding experience (Gupta et al., 2010). Most importantly, Dragoi and Tonegawa (2011) recently reported evidence for pre-play. They recorded spiking activity in CA1 during a period of awake-rest and subsequent running on a linear track. The sequence in which neurons were active during the pre-rest period was predictive of the sequence of the neurons’ place fields on the linear track. This was found even in animals that were exposed to a linear track for the first time. Hence, it appears that the sequence of OSA is intrinsic to the hippocampal network rather than imprinted by external stimuli.

### LACK OF INTERFERENCE IN CA3 WHEN DG-MEDIATED PATTERN SEPARATION IS ABSENT

In the standard framework, DG maps similar input patterns onto uncorrelated attractor states in CA3 to avoid interference between similar memories (pattern separation), a role that is well supported by experimental results. For instance, NMDA receptors in DG (McHugh et al., 2007) were found to be required for the animal to distinguish two similar contexts in contextual fear conditioning. Gilbert and colleagues showed that rats with lesions of the entire hippocampus (Gilbert et al., 1998) or only DG (Gilbert et al., 2001) had deficits in remembering a rewarded location if a lure was close-by (similar spatial patterns), but not if the lure was located far away (distinct spatial patterns). A functional magnetic resonance imaging (fMRI) study in humans reported higher dissimilarity in CA3/DG (they could not resolve CA3 and DG) bold signal than in CA1 (Bakker et al., 2008) when subjects were shown similar visual stimuli. Additional evidence comes from studies that link pattern separation to adult neurogenesis in DG. For instance, pattern separation of visual stimuli (Clelland et al., 2009) and contextual discrimination (Tronel et al., 2012) are impaired after ablation of adult neurogenesis, and improved after an increase in the rate of adult neurogenesis (Creer et al., 2010; Sahay et al., 2011). A recent study goes so far as to suggest that pattern separation of visual stimuli might be supported exclusively by adult-born DG granule cells (Nakashiba et al., 2012).

The interpretation of these studies, however, ignores the purpose of DG-mediated pattern separation in the standard framework: to avoid interference between memories in CA3. First, when a new memory pattern is encountered that is similar to a previously stored pattern, without DG-mediated pattern separation, interference should impede the storage of the new memory and degrade the previously stored memory. While animals could not learn new associations that were similar to old ones in experiments that lesioned DG or inactivated NMDA receptors in DG, the learning attempt did not interfere with the previously stored memories (Gilbert et al., 2001; McHugh et al., 2007). Second, orthogonalization (pattern separation) occurring in the DG does not appear to lead to pattern separation in CA3. In fact, the population vector of place cells in CA3 in similar environments is more similar than that in DG (Leutgeb et al., 2007). Third, if adult neurogenesis were the neural substrate for pattern separation then interference between similar patterns should arise in the absence thereof. This prediction is, however, inconsistent with the properties of adult neurogenesis and with suggestions that DG adult neurogenesis might not even be important for memory formation (Leuner et al., 2006). For instance, the rate of adult neurogenesis is almost 0 in bats (Amrein et al., 2007) who have excellent spatial memory (Tsoar et al., 2011), it is highly variable even between related species (Amrein et al., 2004) and it decreases dramatically with age before the onset of senescence (Seki and Arai, 1995; Kuhn et al., 1996; Gould et al., 1999; Leuner et al., 2007; Knoth et al., 2010). In summary, while we do not disagree that DG might be involved in pattern separation, we see little evidence that DG-mediated pattern separation prevents interference between similar input patterns as hypothesized in the standard framework.

### THE MISSING FUNCTION OF CA1

The standard framework does not offer a strong hypothesis for CA1, but some authors have suggested that it plays a role as novelty or mismatch detector. CA1 is thought to compare the input from entorhinal cortex (EC) III to the retrieved pattern in CA3 and signal any mismatches (Levy, 1989; Hasselmo et al., 1996; Lisman and Otmakhova, 2001; Vinogradova, 2001). When a novel stimulus is encountered, the input will not match any stored pattern. CA1 detects this mismatch and signals novelty. Indeed, CA1 activity increases when rats are exposed to novel environments (Nitz and McNaughton, 2004; Csicsvari et al., 2007; Karlsson and Frank, 2008). Even so, mismatch detection does not seem to be the primary function of CA1. When one of the two main inputs to CA1 is disrupted, mismatch detection is not possible. Since the vast majority of hippocampal outputs pass through CA1, we would expect that interfering with CA1’s primary function would have a similar behavioral effect as removing the entire hippocampus. In contrast, when the input from either CA3 or EC III to CA1 were interrupted (Brun et al., 2002; Remondes and Schuman, 2002; Steffenach et al., 2002; Nakashiba et al., 2008; Suh et al., 2011), the memory deficits were less general than they are after lesions of the entire hippocampus (Morris et al., 1982; Kirwan et al., 2005).

In summary, even though the standard framework has been very influential in the field and is supported by considerable experimental evidence, the evidence against it is mounting, warranting a search for an alternative theory.

## CRISP: A NEW THEORY FOR HIPPOCAMPAL FUNCTION

We posit that episodic and semantic memory differ qualitatively in their neural representation and that hippocampus and neocortex are optimized for storing episodic and semantic memory, respectively. Episodic memories are represented by *sequences* of neural activity patterns, which we will denote as (*u*_1_,…,*u*_*T*_). While some previous studies have modeled episodic memories as sequences (Levy, 1996; Lisman, 1999), it is by far not universally accepted since some studies model episodic memories as static memory patterns (Treves and Rolls, 1992; Hasselmo et al., 2002; Kali et al., 2004). The function of the hippocampus, in our view, is to facilitate the rapid storage and retrieval of neural sequences. Semantic memories, on the other hand, are represented by static patterns of neural activity in the neocortex. While semantic memories may contain similar information as episodic memories, they do not include the same temporal sequence nature as episodic memories.

**FIGURE 1:**
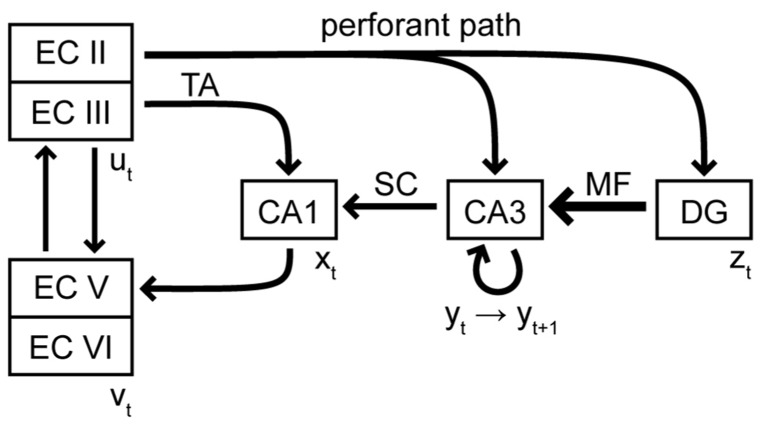
**Schematic of the excitatory connectivity between subregions of the hippocampal formation**. EC, entorhinal cortex; TA, temporoammonic pathway; SC, Schaeffer collaterals; MF, mossy fibers; DG, dentate gyrus. The vectors *u*, *v*, *x*, *y*, and *z* represent the activity pattern at a given time in the appropriate subregion. The arrangement of the subregions emphasizes the hierarchical stacking of CA1, CA3, and DG.

In our view, the hippocampus is therefore always required for the retrieval of true episodic memories, no matter how remote. Episodic memories are not copied to neocortex during systems consolidation, but rather gradually extracted into semantic memories with repeated retrievals. Such as process has been suggested before in the multiple memory trace (MMT) theory (Nadel and Moscovitch, 1997; Nadel et al., 2000). Our theory significantly expands on it. First, we suggest that episodic and semantic memories are not only different in content but also in the nature of their neural representation (sequence vs. static pattern). Second, we suggest specific neural mechanisms for episodic memory storage in the hippocampal subregions and their synaptic connections. We hypothesize that each subarea of the hippocampus, i.e., CA1, CA3, and DG, forms a distinct module that performs a specialized function. These modules are stacked hierarchically, such that each module adds a new functionality to the network (**Figure 1**).

### PATTERN COMPLETION IN CA1

**FIGURE 2:**
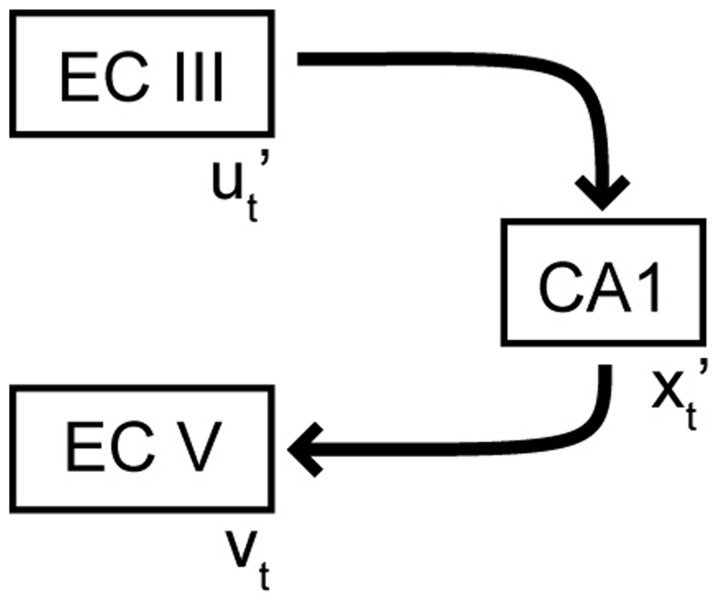
**Pattern completion in the EC–CA1–EC network**. Variables with a prime represent noisy or partial versions of activity patterns.

During storage of an episodic memory, an input pattern in EC, denoted by *u*_*t*_, is associated with a certain pattern in CA1, denoted by *x*_*t*_ . That means the hetero-association between *u*_*t*_ and *x*_*t*_ is stored in the feedforward perforant path synapses, such as in the Willshaw model (Willshaw et al., 1969). At the same time, the output in EC is clamped to the pattern *v*_*t*_ via synaptic connections from the superficial to the deep layers of EC. Hebbian plasticity at the CA1–EC synapses can establish hetero-associations from *x*_*t*_ to *v*_*t*_ . Put together, the feedforward network from EC III to CA1 to deep EC can perform pattern completion, as has been shown to occur in hetero-associative memory models (Willshaw et al., 1969). When a partial cue $u_{t}^{\prime }$ is provided as input, the feedforward EC–CA1 network memory can retrieve the memory pattern $x_{t}^{\prime }$ in CA1 and subsequently the entorhinal output pattern *v*_*t*_ (**Figure 2**). Set up in this way, the EC–CA1–EC loop stores hetero-associations between “static” patterns, i.e., patterns that represent inputs available at the same instant of time. To store episodic memories, we need to store sequences of patterns that extend across time; we next turn to the issue of storing sequences.

### INTRINSIC SEQUENCES IN CA3

**FIGURE 3:**
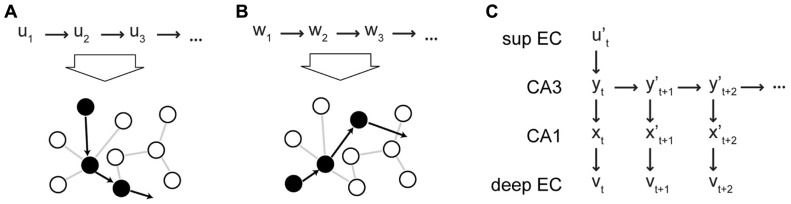
**Intrinsic sequences in CA3 in the CRISP theory**. **(A,B)** Schematic illustration of how different input sequences (shown at the top of the figure) are associated with intrinsic sequences in CA3. The circles represent subpopulations of CA3 neurons. The filled circles represent subpopulations that are activated in the sequences as indicated by the arrows. The sequences of inputs are associated with the intrinsic sequences via plasticity at the EC II–CA3 synapses. **(C)** Retrieval of a stored memory sequence from CA3 based on a partial input cue. The retrieved elements (patterns) are noisy and are cleaned up by the CA1–EC network. The output is fed back to the input to correct the state of CA3 (not shown). sup EC, superficial EC.

Due to its recurrent collaterals, CA3 can generate neural sequences intrinsically, i.e., without external inputs, and this recurrent dynamics allows the hetero-association between successive states *y*_*t*_ → *y*_*t*+*n*_ , where n ≥ 1. We assume that a pool of intrinsic sequences is established in the network during development. Plasticity at the recurrent collaterals in CA3 plays a role in this process and in maintaining the pool of intrinsic sequences (which might be challenged by synaptic changes due to noisy activity and homeostatic processes). However, we posit that recurrent synapses remain relatively fixed during memory storage. Instead, we posit that plasticity in the feedforward synapses to CA3 is critical for sequence storage. To store a sequence (*u*_1_,…,*u*_*T*_) that represents an episodic memory, an intrinsic sequence (*y*_1_,…,*y*_*T*_) is activated in CA3 (by context reset from the DG, see next paragraph) and each element *u*_*t*_ is associated with a particular CA3 state y_t_ . This association occurs via Hebbian plasticity at the perforant path synapse from EC II to CA3 (**Figures 3A,B**). The activation of the CA3 sequence depends on the DG and is described below. At the same time, the CA3 state y_t_ is also linked via the Schaeffer collaterals with the CA1 pattern *x*_*t*_ , which in turn is associated with the output pattern *v*_*t*_ as described above. We suggest that these downstream associations make the retrieval of sequences more robust. Some errors might be introduced by noisy spiking, spiking failures, or errant inputs during sequential retrieval from CA3, i.e., y_t_ leads to a noisy version $y_{t+1}^{\prime }$ of the correct pattern *y*_*t*+1_. Since these errors would accumulate and make the recall of an extended sequence noisy (cf. Lisman, 1999), they need to be corrected. In our theory, the pattern completion network in CA1 and EC can retrieve the correct output pattern *v*_*t*+1_ associated with *y*_*t*+1_ (**Figure 3C**). This pattern can be fed back to CA3 via EC V to EC II to initiate the recall of the next item. As a result, the combined EC–CA3–CA1–EC system can retrieve an extended sequence based on a single partial element $u_{t}^{\prime }$.

### CONTEXT RESET BY DG

**FIGURE 4:**
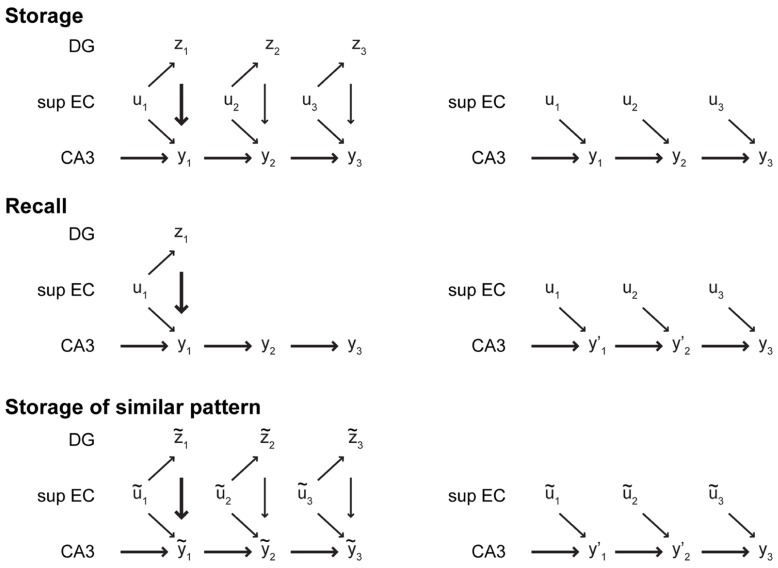
**Illustration of the role of DG in memory storage and recall**. *Left column*: During storage and recall in the intact hippocampus, DG initially drives CA3 strongly (thick arrows), forcing it to change its state that otherwise is mostly driven by the intrinsic dynamics of CA3 (medium arrows). Subsequently, the DG drive weakens (thin arrows), allowing CA3 to express an intrinsic sequence. During storage, the input patterns *u*_*t*_ are associated with the CA3 states y_t_ , which can be recalled later based on some cue. If a new episode is similar to a previously stored sequence, denoted by (*ũ*_1_,…,*ũ*_*T*_), the initial DG drive can select a different intrinsic sequence to form a new episodic memory. *Right column*: In the absence of the DG, two deficits may appear. First, without the strong initial drive from DG, the intrinsic dynamics of CA3 resist the inputs from EC. $y_{t}^{\prime }$ denotes a noisy or incorrect version of the correct CA3 state. A sequence of consistent inputs might eventually bias the CA3 network enough to retrieve the correct sequence. Second, without pattern separation by the DG, exposure to a similar episode triggers recall of the previously stored sequence rather than encoding of a novel episodic memory.

At least two properties of the CA3–CA1 system described so far need further attention. First, the CA3 recurrent dynamics impedes the initiation of cue-driven recall. Since episodic memories are stored via association of external inputs with CA3 sequences that are generated intrinsically, CA3 has to be driven more strongly by its recurrent dynamics than by its synaptic inputs from EC II. This relationship has been observed previously (Urban et al., 2001). Second, the CA1–CA3 network, favors pattern completion and thus cannot distinguish between similar sequences. In our view, the DG could solve both problems with a single mechanism: context reset. When a novel input is encountered, i.e., a new episode needs to be encoded or recall needs to be initiated, input from DG resets the CA3 recurrent dynamics (thick arrows in **Figure 4**). Specifically, we suggest that through the exceptionally strong mossy fiber synapse (some authors call it a “detonator synapse”) DG drive can interrupt the ongoing CA3 sequence and initiate a new intrinsic CA3 sequence.

Once the new sequence has been initiated in CA3, the DG drive has to weaken to allow the CA3 recurrent dynamics to generate the intrinsic sequence either for recalling a stored sequence or for encoding a novel one (thin arrows in **Figure 4**). Several unique properties of the mossy fiber synapse might serve as mechanism for modulating the strength of DG drive. For instance, DG inputs are more likely to drive downstream CA3 spike, if the DG cell spikes at a high rate (Urban et al., 2001; Henze et al., 2002; Mori et al., 2004) and there are suggestions that firing rates are higher in novel than in familiar environments, at least in CA1 (Karlsson and Frank, 2008). Another potential mechanism is the depression of synaptic transmission at the mossy fiber synapse with increased number of spikes in a train (Mori et al., 2004). Finally, a potential network level mechanism could be provided by acetylcholine that might modulate the subregions of the hippocampus differentially, increasing synaptic currents in DG and decreasing the strength of CA3 recurrent connections (Hasselmo, 1999).

The associations between the DG pattern z_t_ and the EC input *u*_*t*_ can be stored in the feedforward EC–DG network, which might be similar to the EC–CA3 and EC–CA1 networks. The EC–DG network can therefore pattern complete and hence make retrieval more robust to noise. However, this pattern completion also counteracts pattern separation during storage of novel inputs. As a solution to this dilemma, we suggest that adult-born granule cells are integrated into the DG network to improve pattern separation. Since newborn cells have lower thresholds for excitation and plasticity (Snyder et al., 2001; van Praag et al., 2002; Schmidt-Hieber et al., 2004), we hypothesize that they are more likely to become active when a novel input is encountered. Newborn cells also establish new synaptic connections and thus might project to CA3 cells that do not receive any of the sparse Mossy fiber connections, thus further aiding in pattern separation.

## EXPERIMENTAL SUPPORT FOR THE CRISP THEORY

### MULTIPLE MEMORY SYSTEMS AND SYSTEMS CONSOLIDATION

In CRISP, the fundamental difference between cortex and hippocampus is the nature of the represented content. The hippocampus stores sequential episodic memories, neocortex stores static semantic memories. This view is supported by experimental evidence. The hippocampus was found to be involved in sequence learning (Lehn et al., 2009) and in tasks that require manipulation of associations across time or space. For instance, the hippocampus is needed for learning transitive interference (Bunsey and Eichenbaum, 1996; Dusek and Eichenbaum, 1997) and fMRI studies show that transitivity activates the human hippocampus (Heckers et al., 2004; Preston et al., 2004) and that the activity is correlated with the size of the discontiguity (Staresina and Davachi, 2009). After episodic memories are stored in the hippocampus, we propose that semantic information is extracted and stored in neocortex. This process could be similar to slow-feature analysis, which can extract invariant features from a time varying input stream (Wiskott and Sejnowski, 2002). Since this process requires repetition, episodic memories have to be reactivated or recalled repeatedly to become incorporated into semantic memory. This process has been called semanticization. Since more remote episodic memories are more likely to have been recalled repeatedly, they are more likely to have been semanticized and thus to be independent of the hippocampus. Our theory can therefore account for graded retrograde amnesia.

In addition, our theory is also consistent with several observations regarding system consolidation that are at odds with the standard framework. First, our theory predicts that hippocampal damage affects all episodic memories, because even remote episodic memories will be lost (Bolhuis et al., 1994; Martin et al., 2005; Lehmann et al., 2006; Sutherland et al., 2008) if they have not been recalled repeatedly and thus semanticized. Second, true episodic memories always require and activate the hippocampus (Nadel et al., 2000; Steinvorth et al., 2005; Harand et al., 2012). Third, according to our theory there is no fundamental difference in the relative speeds of memory formation in neocortex and hippocampus (Murray and Mishkin, 1985; Vargha-Khadem et al., 1997; Cipolotti et al., 2006; Bird et al., 2008; Sharon et al., 2011), but rather a difference in the timescale at which the stored information arises. Episodic memory inherently has a one-shot-learning character, while the semanticization of memories involves the synthesis of information from across multiple experiences.

### PATTERN COMPLETION IN CA1

Our hypothesis that CA1 performs completion of multi-modal patterns leads to several implications that can be compared to experimental results. First, some hippocampally dependent tasks can be supported by the EC–CA1–EC circuit alone in the absence of CA3 and DG. Note that this is different from episodic memory, since a static pattern does not have the essential quality of episodic memory, which in our theory is that they are sequential in time. In fact, behavioral experiments have found that spatial recognition memory is not impaired when CA3 is disconnected from the network (Brun et al., 2002). Learning in the Morris water maze, which requires the hippocampus as a whole (Morris et al., 1982; Kirwan et al., 2005), is preserved after synaptic transmission from CA3 to CA1 is genetically disabled (Nakashiba et al., 2008). Other CA3 disruptions studies found only a small deficit in water maze learning (Brun et al., 2002; Steffenach et al., 2002). Second, since pattern completion in CA1 is used for error correction during retrieval of extended temporal associations, we expect that errors accumulate if EC III inputs to CA1 were removed, as reported recently by Suh et al. (2011). Third, the converse implication is that EC III to CA1 projections should not be required for retrieval that does not bridge longer time intervals. This expectation is supported by findings that animals can still learn the reference version of the water maze task (Remondes and Schuman, 2004; Suh et al., 2011) and contextual fear conditioning (Suh et al., 2011) without EC III inputs to CA1.

The proposed role of CA1 leads to an experimentally testable prediction. Since CA1 is hypothesized to receive the same, albeit sometimes corrupted, inputs via both pathways, place fields in CA1 driven by CA3 alone and driven by EC III alone should be in the same location. Indeed, there are reports that CA1 place fields in familiar environments look very similar to controls when either CA3 (Nakashiba et al., 2008) or EC III (Suh et al., 2011) inputs are removed. However, it remains unknown whether the CA1 place fields are in the same locations in both cases. To address this question, one could temporarily inactivate one of the two inputs at a time, for instance, with optogenetic methods, and compare the place fields of a given CA1 cell in these two and the control conditions.

### PLASTICITY IN CA3

Our theory is more consistent with experimental studies on changes in CA3 place cell responses than the standard framework is. In our theory, since CA3 neurons are activated by intrinsic dynamics, the sequence of their activation remains relatively fixed. As a result, place fields in CA3 would change relatively little during memory formation. This pattern has been observed in experiments in which animals learn about a novel environment, as reviewed in Section “Plasticity in the Recurrent CA3 is Not Necessary for Rapid Learning.” In particular, we refer to observations that the locations of place fields in CA3 do not change during a learning task, while those of simultaneously recorded CA1 cells do (Dupret et al., 2010).

Future experimental studies are needed to test our ideas regarding the function of synaptic plasticity in CA3. To associate sequences of neocortical inputs with intrinsic sequences in CA3, as necessary in our theory, plasticity at the EC II–CA3 synapse is critical at the moment of memory storage. While plasticity at the recurrent CA3 synapses plays no role in memory storage in our theory, it might be required on a longer time scale to maintain or fine-tune the intrinsic sequences. Tests of these predictions face challenging requirements. First, plasticity has to be inactivated transiently and selectively at either the EC II–CA3 or the recurrent CA3 synapse. Second, animals have to be tested on memory tasks that have been demonstrated to require CA3. As discussed in Section “Pattern Completion in CA1,” we suspect that many hippocampally dependent tasks can be supported by the EC–CA1–EC loop in the absence of CA3. To our knowledge, such studies have not been performed, yet. While experimental studies have reported long-term potentiation (LTP) at the EC II–CA3 synapse (Do et al., 2002), its functional role has received little attention. Genetic manipulations of NMDA receptors in CA3 targeted both EC II–CA3 and recurrent CA3 synapses (Nakazawa et al., 2002, 2003; Cravens et al., 2006) and it remains unclear which is responsible for the observed behavioral deficits. The interpretation is further complicated by another observation: EC II–CA3 synapses exhibit NMDA-independent LTP in addition to NMDA-dependent LTP (Do et al., 2002). Finally, the genetic manipulation of CA3-NMDA receptors was chronic and thus would affect the hypothesized maintenance mechanism and indirectly memory storage and retrieval as well. We conclude that the inconsistent findings in CA3-NMDA mutant mice might stem from confounding the several processes mentioned above. In our opinion, more studies are required to clarify the specific role of plasticity at the EC II–CA3 and recurrent CA3 synapse.

### INTRINSIC SEQUENCES IN CA3

In line with recent experimental results, OSAs are the product of intrinsic CA3 dynamics in the CRISP theory. Intrinsically generated neural sequences have been observed in the hippocampus (Pastalkova et al., 2008; MacDonald et al., 2011); they are precisely timed (Itskov et al., 2011), and in one case represent time in a sequence rather than the item itself (Naya and Suzuki, 2011). If CA3 is more strongly driven by its intrinsic dynamics than by external inputs at some point in time, then this would imply that memory performance depends on the state of the hippocampus preceding the stimulus presentation, as observed recently (Park and Rugg, 2010).

Unlike the standard framework, in which input sequences are stored, the CRISP theory can account for pre-play thanks to the intrinsic sequences in CA3. In the offline state, the neural network in CA3 is driven by noise and generates sequences randomly. Due to the strong network dynamics, some intrinsic sequences will be activated. When the animal enters a novel environment later, the sensory inputs are mapped onto some of those intrinsic sequences. As a result, the order of OSA during pre-sleep becomes predictive of the order of CA3 place fields recorded later. Since pre-play has been reported only in CA1 to date, we hypothesize that CA3 passes OSA on to CA1.

### THE FUNCTIONAL ROLE OF THE DENTATE GYRUS AND ADULT NEUROGENESIS

Our theory assumes that DG overrides the strong intrinsic CA3 dynamics during the initiation of memory storage and retrieval, in contrast to the standard framework, which argues against a role of the DG in retrieval (Treves and Rolls, 1992). We conjecture that the DG override accelerates the retrieval process compared to retrieval through the weaker EC II–CA3 pathway (**Figure 4**). Both theories are therefore consistent with observations that DG projections to CA3 are not required for retrieval when sufficient time is available (Lassalle et al., 2000; Lee and Kesner, 2004). But only our theory can account for the results of a recent study, in which synaptic outputs of mature granule cells were silenced genetically (Nakashiba et al., 2012). Like in the earlier studies, mutant mice had no deficits in recall when given sufficient time (3 min), but they had significant deficits in initiating recall rapidly (within 10 s). There is more direct evidence for a competition between the DG signaling a novel context and CA3 intrinsic dynamics resisting the switch. When Jezek et al. (2011) suddenly switched the context from one to another, the spatial representation encoded by place cells flickered between the distinct representations of the two contexts. We predict that this switch would take longer than the observed single theta cycle if DG were removed from the network.

Since DG-mediated pattern separation ensures that novel inputs that are similar to established memories are stored as new memories, our theory relies on pattern separation in DG. It is therefore consistent with experimental results that support a pattern separation function of DG (see Lack of Interference in CA3 When DG-mediated Pattern Separation is Absent). The critical difference to the standard framework is that CRISP does not require pattern separation to avoid interference in CA3. Without pattern separation, e.g., when DG is lesioned, similar input patterns would be associated with the same intrinsic sequence in CA3. The animal would therefore be unable to distinguish between the similar inputs (**Figure 4**), but no interference occurs because no new memory pattern is stored in CA3. Our theory thus accounts for the lack of interference in pattern separation experiments (Gilbert et al., 2001; McHugh et al., 2007) and the overwhelming experimental evidence that adult neurogenesis is not required for memory storage *per se*.

We hypothesize that adult neurogenesis benefits a species or individual only if their survival depends on exhibiting different behaviors in similar situations. This might be important for young individuals with little prior experience, for animals that face strong pressure from predators, and for animals living in rapidly changing conditions. By contrast, other species or individuals might benefit from more stereotyped behavior in subtly different situations. For example, older individuals with a large repertoire of prior experience, animals with no or few competitors, and animals that live in relatively constant environments. This view is consistent with the large variability of the rate of adult neurogenesis across age (Seki and Arai, 1995; Kuhn et al., 1996; Gould et al., 1999; Leuner et al., 2007; Knoth et al., 2010), individuals (Gatome et al., 2010), and species (Amrein et al., 2007). We predict a correlation in mammals between the flexibility of their natural behavior and the rate of adult neurogenesis in the DG.

## COMPARISON TO OTHER THEORIES

Due to space constraints we will limit ourselves to discussing a sample of theories that span the range of theories in the literature. Our theory is closely related to MMT theory, which was conceived to explain systems consolidation (Nadel and Moscovitch, 1997; Nadel et al., 2000) and hypothesizes that neocortex and hippocampus store parallel, but different, traces of a memory. Traces are established first in the hippocampus and with each retrieval another trace of the original memory is created in the neocortex. Our theory builds on MMT theory and suggests specific neural implementations of episodic memory storage and retrieval. Both theories thus share many predictions about the behavioral implication of systems consolidation (see Multiple Memory Systems and Systems Consolidation), but our theory can make predictions for electrophysiological results and the behavioral effects of specific network manipulations that are outside the scope of MMT theory.

The spatial hypothesis for the hippocampus argues that, unlike in humans, the hippocampus in rodents is not involved in memory and specialized in representing space (Colgin et al., 2010). Aspects of the spatial hypothesis are well supported by several observations. The stunning discovery of periodic grid cells in the rodent MEC (Hafting et al., 2005), the demonstration that the entire hippocampus, including the ventral aspects, contains spatially selective neurons (Kjelstrup et al., 2008), and the observation that place cells respond to the geometry of the environment (O’Keefe and Burgess, 1996; Lever et al., 2002). While some authors suggest that the hippocampus performs path integration (McNaughton et al., 1991; Samsonovich and McNaughton, 1997), others favor a cognitive map (O’Keefe and Nadel, 1978). However, other results show that the hippocampus is required in rodents for tasks that do not have any overt spatial component such as memory of odor sequences (Fortin et al., 2002), trace eyeblink conditioning to an airpuff (Weiss et al., 1999) or trace fear conditioning to a tone (McEchron et al., 1998). It remains unclear how the spatial hypothesis can account for these results.

Another interesting suggestion is that the hippocampus did not evolve primarily to store memories, but to simulate future events, which are loosely based on past experience (Levy, 1989; Dudai and Carruthers, 2005; Byrne et al., 2007). This hypothesis could explain why our episodic memories are so faulty (Schacter, 2002). There is experimental evidence that hippocampal patients have deficits in mentally constructing imaginary situations (Hassabis et al., 2007), although the experimental evidence is currently mixed (Maguire et al., 2010; Squire et al., 2010). Some authors suggest that the hippocampus is involved only because it puts together (Hassabis et al., 2007) or stores the narrative (Martin et al., 2011). However, even in the mental simulation hypothesis, the hippocampus is involved in the storage of episodic memories; also this hypothesis does not suggest a storage mechanism.

Several other models assume, like we do, that CA3 stores sequences of neural activity patterns (Levy, 1996; Amarasingham and Levy, 1998; Wallenstein et al., 1998; Lisman, 1999) and can naturally explain the involvement of the hippocampus in associating discontiguous items in space or time. Of these models, the one closest to ours is the SOCRATIC (sequences of condensed representations, autocorrected, theta-gamma coded, in context) model (Lisman, 1999; Lisman and Otmakhova, 2001). This model argues that sequences are stored in the combined CA3 and DG network, where DG stores auto-associations (akin to static patterns in our theory) in its complex recurrent network from granule cells to mossy cells, and CA3 stores hetero-associations that link one item in a sequence to the next. The faint backprojection from CA3 to DG sends the retrieved next item in the sequence to be cleaned up by the DG auto-associative network. Several elements are common to both the SOCRATIC model and CRISP, such as the distinction between static patterns vs. sequences, sequences rather than auto-associations in CA3, and a correction mechanism for extended sequences. However, there are also important differences. First, the sequence correction mechanism is provided by the feedforward EC–CA1–EC network in our theory, and by DG in the SOCRATIC model. Second, the SOCRATIC model does not explicitly implemented pattern separation. Third, and perhaps mostly, CA3 sequences are imposed by external inputs and stored by CA3 in the SOCRATIC model. This feature is shared by other models of sequence memory in CA3 and these models therefore suffer from the same difficulties as the standard framework in explaining why blocking plasticity at the recurrent CA3 synapses causes only minor deficits (see Plasticity in the Recurrent CA3 is Not Necessary for Rapid Learning).

Most other alternative explanations of the time-limited effect of amnesic agents in the hippocampus ascribe some memory storage function to the hippocampus. The major distinguishing feature of CRISP is the idea that CA3 recurrent connections do not change on a fast time scale, while the feedforward weights to and from CA3 are highly plastic. The computational advantage of our suggestion is that learning in feedforward networks is far simpler than in recurrent networks (Jaeger and Haas, 2004; Cheng and Frank, 2011) and feedforward memory networks have higher memory capacities than recurrent networks (Palm and Sommer, 1992).

## CONCLUSION

We have proposed a new theory of hippocampal function in episodic memory based on pattern completion in CA1, intrinsic sequences in CA3, and context reset in DG. In this article, we have discussed the conceptual theory, and its implications and predictions for experimental results. We have argued that they are consistent with most major experimental findings, many of which are difficult to reconcile with the standard framework. The power of a theory lies in the way it can drive future work, both modeling and experimental. We have identified a number of predictions that can be tested experimentally and questions that can be studied theoretically. Future work is needed to show that a computational model based on the CRISP theory can store and retrieve memories as envisioned. Quantitative studies are also required to study the importance of anatomical parameters in the hippocampus, such as the recurrent connectivity rate in CA3, for performing the functions postulated by our theory. This and other work may corroborate or falsify our theory, but either way the outcome will advance our understanding of the role of the hippocampus in episodic memory.

## Conflict of Interest Statement

The author declares that the research was conducted in the absence of any commercial or financial relationships that could be construed as a potential conflict of interest.
